# London County Westminster and Parr's Bank Annual Meeting

**Published:** 1920-02-14

**Authors:** 


### London County Westminster and Parr's Bank Annual Meeting.

### A Plea for Economy—Currency Problems.

At the Annual General Meeting of the Shareholders of the London County Westminster & Parr's Bank, held on February 5, Mr. Walter Leaf (the Chairman) analysed the leading figures of the Balance Sheet. They were, he said, truly remarkable figures. The Bank's deposits had grown during the year by no less than 42 millions, of which about six were due to the absorption of the Nottingham and Notts Bank. In so far as the figures were only a reflexion of an inflated currency, these large figures were by no means a ground for satisfaction; and they would be entitled to congratulate themselves when a falling off in circulation began to reduce them to a more modest total. There was a large increase under the head of Advances to Customers and Acceptances. This was the measure of the assistance the Bank was giving to the remarkable increase of British commerce and industry, which was developing, as they had always hoped, with the long-delayed coming of peace. The demands which they anticipated, and which were a strong motive for the strengthening of the large banks by amalgamation, were now coming upon the bank in full force, and at the present rate promised soon to tax all its resources to the full. Referring to our imports and exports, he quoted certain items from the Board of Trade Returns—especially articles of luxury and foreign wines and spirits. Here it was that we should look for an earnest effort of self-denial if we were honestly determined to hold up our heads before the world and face our creditors with a clear conscience. He said it was to his mind a scandal that, when everyone should be earnestly doing his best to put the national balance sheet straight, we should during 1919 have imported no less than £26,695,000 of foreign wines and spirits. Of this, importations from Portugal—that was, port wine—amounted to over eight and a half millions, and champagne alone to £3,861,000. It was an increase over 1918 of sixteen and a half millions—all a dead loss, a hindrance and a discouragement to those who were striving earnestly to set our national finances on a solvent basis. This was only a part of the whole system of wanton extravagance which was only too obvious.

GREAT BRITAIN AND UNITED STATES.

Mr. Leaf said that he had had an opportunity of discussion with many of the leading men, in political thought as well as in finance, alike in New York, Washington, Boston, and Chicago. Everywhere he was impressed with the feeling of real friendliness towards this country. On the other hand, there was clearly a strong revulsion, after the enthusiasm of the war, against European entanglements. Of the traditional boastfulness of our American cousins he saw nothing; the part that this country had played in winning the war was more than generously acknowledged, and he never heard a more eloquent eulogy of England than from the mouth of a Chicago banker who had held an important post, with the rank of Brigadier-General, in the American Army in France. When the talk turned on European mandates, he had rather to complain of American modesty—a reluctance to take any part in European administration on the ground that the United States had no trained Civil Service capable of the work. And it was clear that in this direction no help was to be looked for. When it came to a question of credits, the American attitude was distinctly critical and suspicious of our expenditure on luxury—all the more because they had imposed on themselves the severe restrictions of the Prohibition law. This was continually brought up against him, and he found it difficult to reply. There was also an idea, which he disclaimed without hesitation, that the fall in the American exchange was favourably regarded here as an incentive to our export trade. But, generally speaking, the attitude taken was that which had been so forcibly summed up by Mr. Hoover—that the remedies for our own troubles were in our own hands, in our own resolution to work and save; and that it was neither prudent nor consistent with our dignity as a great nation that we should come to the United States with petitions for great loans to help us through. With that view he was entirely in agreement; and he could not help expressing his regret that well-meaning—but, he was afraid, mistaken—appeals should have been recently made for amounts which could only defeat co-operation by their very magnitude. That was not the policy either of English financiers nor of the British Government. So long as we take the bold stand that we mean to pay our debts, and were well able to do so; and that we were anxious to co-operate with the United States in the great task of the restoration of Europe, he for one had no fear either of the loss of our commercial and financial place in the sun, nor of any serious discord between the two great Anglo-Saxon nations on whose friendship such vast issues depend.

THE CURRENCY QUESTION.

He went on to say, the question of the depreciation of our currency, with its direct effect in the rise of prices, was a matter which came home to all of us every day, and was at the bottom of a great part of the universal sense of unrest. He was no believer in proposals for artificial restrictions of the currency. It would be like trying to cure a fever by plugging your clinical thermometer at "normal," and the only result would be that you would burst your thermometer. The only remedy was to attack the fever, to stop the issue of claims to currency in the form of Government expenditure and Government credits.

INTERNATIONAL POSITION.

Finally, he alluded to the International position, observing that it was necessary to take a wide and humane view, a generous outlook which took cognizance of more than the passions of the moment. We must learn to put aside all thoughts of mere destruction, and to recognise that the attempt to annihilate Germany by preposterous demands for reparation would hurt ourselves far more than it will hurt Germany, and would mean not only economic but social suicide for England. Nor must there be any boycott of German trade. The blackness of the cloud which hung over the Continent was alarming to us all, and was a gloomy background to all the hopes which we could base upon our own reviving trade. But the prospect must be boldly faced and fearlessly handled; and we might well hope that the coming year might see, abroad as well as at home, the turning-point of our economic troubles with our own land in unquestioned place as the leader in the new civilisation.

The Report was unanimously adopted and other formal business transacted. A hearty vote of thanks to the Chairman closed the proceedings.

